# An *In Vivo* Metabolic Approach for Deciphering the Product Specificity of Glycerate Kinase Proves that Both *E*. *coli’s* Glycerate Kinases Generate 2-Phosphoglycerate

**DOI:** 10.1371/journal.pone.0122957

**Published:** 2015-03-30

**Authors:** Lior Zelcbuch, Manuel Razo-Mejia, Elad Herz, Sagit Yahav, Niv Antonovsky, Hagar Kroytoro, Ron Milo, Arren Bar-Even

**Affiliations:** 1 Department of Plant and Environmental Sciences, Weizmann Institute of Science, Rehovot Israel; 2 Department of Biochemistry and Molecular Biophysics, California Institute of Technology, Pasadena, California, United States of America; 3 Max Planck Institute of Molecular Plant Physiology, Potsdam-Golm, Germany; University of Freiburg, GERMANY

## Abstract

Apart from addressing humanity’s growing demand for fuels, pharmaceuticals, plastics and other value added chemicals, metabolic engineering of microbes can serve as a powerful tool to address questions concerning the characteristics of cellular metabolism. Along these lines, we developed an *in vivo* metabolic strategy that conclusively identifies the product specificity of glycerate kinase. By deleting *E*. *coli*’s phosphoglycerate mutases, we divide its central metabolism into an ‘upper’ and ’lower’ metabolism, each requiring its own carbon source for the bacterium to grow. Glycerate can serve to replace the upper or lower carbon source depending on the product of glycerate kinase. Using this strategy we show that while glycerate kinase from *Arabidopsis thaliana* produces 3-phosphoglycerate, both *E*. *coli*’s enzymes generate 2-phosphoglycerate. This strategy represents a general approach to decipher enzyme specificity under physiological conditions.

## Introduction

The identification of the *in vivo* substrates of enzymes can be, in some cases, a non-trivial challenge. In fact, numerous enzymes, most belonging to secondary metabolism, were assigned a new catalytic role after further studies shed light on their actual role *in vivo*, *e*.*g*., [1–4]. More uncommon are cases in which the substrate itself is known but the residue on which the enzyme acts is unclear. *E*. *coli*’s glycerate kinase enzymes present such an interesting case in central carbon metabolism. During the last decades several studies on the bacterium’s two glycerate kinases, encoded by *garK* and *glxK*, were published. Earlier *in vitro* assays claimed that these enzymes generate 3-phosphoglycerate [5,6] while more recent studies, using advanced experimental methodologies, provided evidence that 2-phosphoglycerate is the product of the enzymes [7,8]. In fact, even the physiological role of the two enzymes seems to be disputed. For example, while it was claimed that glxK is necessary for growth on glycolate [9] we found that a deletion of *glxK* resulted in no clear phenotype when glycolate, glyoxylate or glycerate are provided as sole carbon sources. On the other hand, deletion of *garK* led to a severe retardation of growth using these substrates.

Although the *in vitro* experiments supporting 2-phosphoglycerate as being the product of *E*. *coli*’s glycerate kinase enzymes are quite compelling (see discussion in [8]), we reasoned that this metabolic conundrum could also be addressed by using an *in vivo* metabolic selection strategy. By employing such a metabolic engineering approach we unequivocally demonstrate that the product of both glycerate kinase variants is indeed 2-phosphoglycerate, thereby suggesting that a glycerate 3-kinase activity is missing in *E*. *coli*.

## Results & Discussion

**Fig 1:**
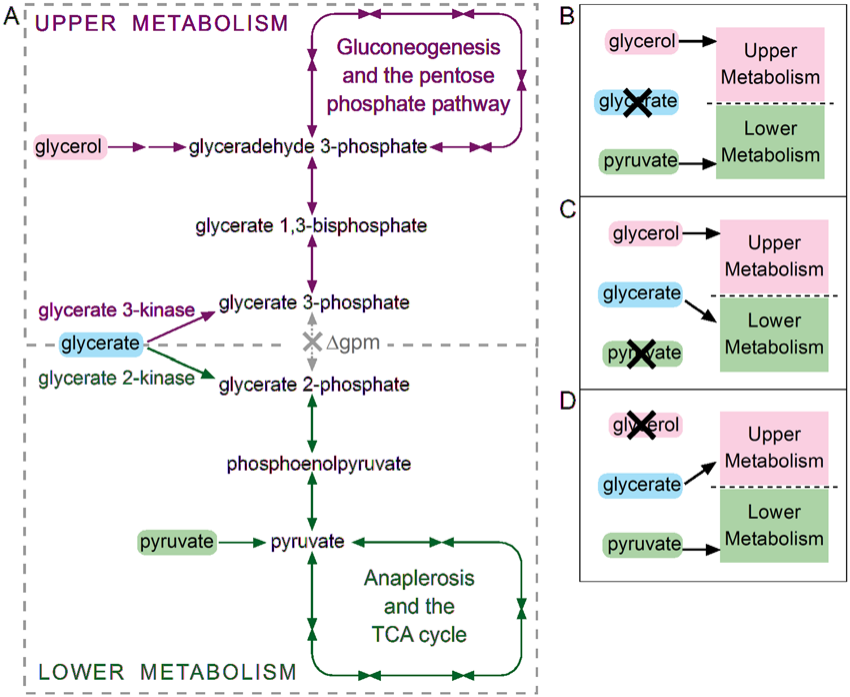
Selection scheme for identifying the product specificity of glycerate kinase. (A-D) By deleting phosphoglycerate mutase, central metabolism is divided into ‘upper’ and ‘lower’ metabolism, each requires its own carbon source for the bacterium to grow (glycerol and pyruvate, respectively). Glycerate can replace one of these carbon sources, depending on the product specificity of glycerate kinase.

To resolve the product specificity of garK and glxK we developed an *in vivo* metabolic assay that identifies the enzyme product via a simple growth selection experiment. We started by deleting the two endogenous phosphoglycerate mutase enzymes (Δ*gpmA*, Δ*gpmM*). As far as we know, this is the first time such a double knockout mutant was generated [10]. The central metabolism of this strain is effectively cut into ‘upper’ and ‘lower’ metabolism, as shown in Fig 1A. For this strain to grow on a minimal medium, it should be supplied with two carbon sources (Fig 1B), an ‘upper’ one, *e*.*g*., glycerol, and a ‘lower’ one, *e*.*g*., pyruvate (for an analogous approach in a completely different context see refs. [11,12]). The Δ*gpmA* Δ*gpmM* strain grew on glycerol and pyruvate at a doubling time of ~120 minutes. We further deleted *garK* and *glxK* to establish a glycerate kinase-free background. This background enables a direct selection for glycerate 2-kinase and 3-kinase activities by modulating the growth medium: glycerate can replace the lower carbon source if glycerate 2-kinase activity is present (Fig 1C); alternatively, glycerate can replace the upper carbon source if glycerate 3-kinase activity is present (Fig 1D).

**Fig 2:**
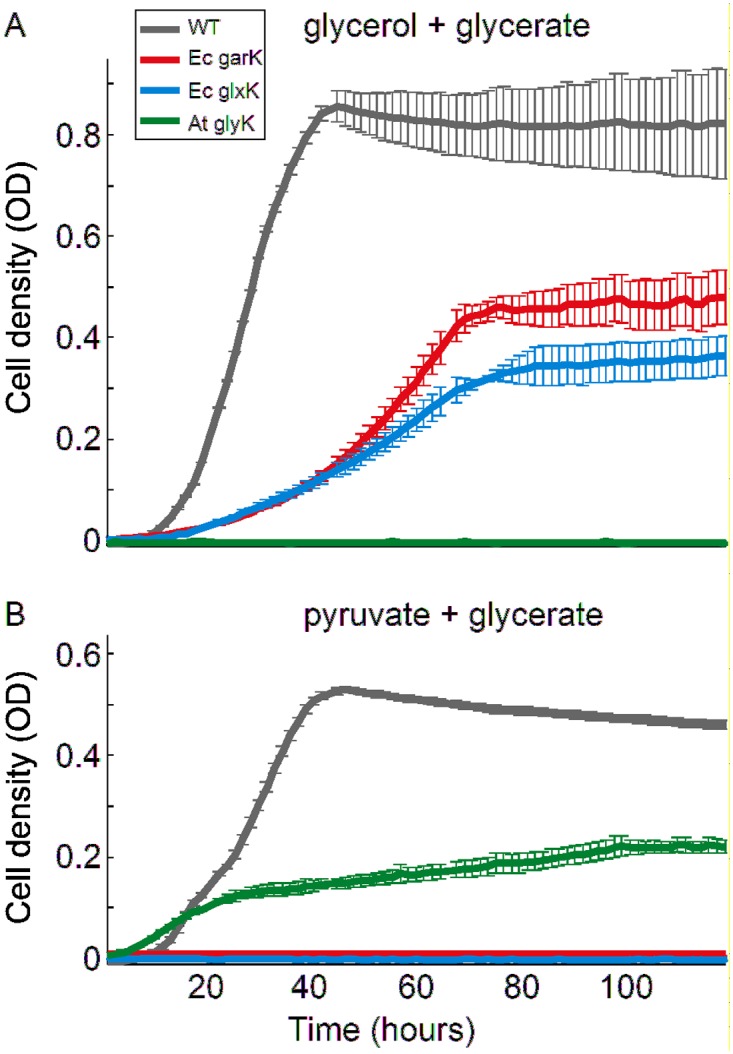
Product specificity of glycerate kinases from *E. coli* and A. thaliana. (A) A *ΔgpmA ΔgpmM ΔgarK ΔglxK* strain was able to grow on a minimal medium supplemented with glycerol and glycerate when one of *E. coli’s* glycerate kinases were expressed, indicating that these enzymes generate 2-phosphoglycerate. (B) Growth on pyruvate and glycerate was possible only when *A. thaliana’s* glycerate kinase was expressed, indicating that this enzyme produces 3-phosphoglycerate. Cells were cultivated in 96-multiwell plates and OD measurements were taken automatically every 90 minutes. For each enzyme, we show the growth of one clone with standard errors (at each time point) that are based on three parallel cultivations. Other clones showed a similar qualitative dependence on the carbon sources with somewhat different growth yield and dynamics.

We tested three glycerate kinases by overexpressing them in the above strain: *E*. *coli*’s protein products of *garK* (Ec garK) and *glxK* (Ec glxK), as well as *Arabidopsis thaliana*’s glycerate kinase (At glyK), which is known to produce 3-phosphoglycerate [13]. The results are shown in Fig 2 and summarized in Table 1. The expression of Ec garK or Ec glxK enabled glycerate to replace pyruvate as a carbon source, while At glyK expression enabled glycerate to replace glycerol as a carbon source. The WT strain displays a considerable yield difference between growth on glycerol + glycerate and growth on pyruvate + glycerate, probably due to the fact that glycerol is considerably more reduced than pyruvate [14]. This is also reflected in yields of the Δ*gpmA*, Δ*gpmM* strains, which were about half of the WT strain (Fig 2).

**Table 1:** Summary of selection experiments on a minimal medium supplemented with different carbon sources.

Carbon sources	Expressed enzymes		
	Ec garK	Ec glxK	At glyK
Glycerol	−	−	−
Pyruvate	−	−	−
Glycerate	−	−	−
Glycerol + Pyruvate	+	+	+
Glycerol + Glycerate	+	+	−
Pyruvate + Glycerate	−	−	+

Different glycerate kinase variants were expressed in the ΔgpmA ΔgpmM ΔgarK ΔglxK strain and growth phenotypes on different carbon sources were measured. A ‘-’ sign corresponds to experiments that showed no growth, while a ‘+’ sign corresponds to growth. Cells were cultivated in 96-multiwell plates using an automated robotic platform (exact experimental setup is given in Materials and Methods).

These finding clearly demonstrate that while At glyK is indeed specific to 3-phosphoglycerate, both *E*. *coli*’s glycerate kinases produce 2-phosphoglycerate. Notably, current metabolic models of *E*. *coli* suggest that the bacterium has a glycerate 3-kinase enzyme (*e*.*g*., ref. [15]); these models should be amended according to the findings we show here.

What is the physiological difference between phosphorylating glycerate at the alpha position and the beta position? We speculate that the main difference is energetic. The proximity of the negatively charged phosphate to the negatively charged carboxyl in 2-phosphoglycerate makes it more energetic than 3-phosphoglycerate (*i*.*e*., having higher Gibbs energy of formation). In fact, the conversion of 3-phosphoglycerate to 2-phosphoglycerate has Δ_r_G’° > +6 kJ/mol under *E*. coli’s physiological conditions of pH 7.5 and ionic strength of 0.25 M [16]. As the glycerate kinase reaction dissipates a lot of energy regardless of the exact product it generates (Δ_r_G’° < -18 kJ/mol, under the same conditions), it makes perfect sense for the cell to produce the more energetic compound that can be converted to its counterpart favorably. If 3-phosphoglycerate was the kinase’s product, its conversion to 2-phosphoglycerate and its downstream metabolites will suffer from a reduced thermodynamic driving force due to the energetic barrier [17]. A notable exception is when almost all the flux is channeled in the gluconeogenesis direction, as is the case of the glycerate kinases that participate in plant photorespiration [18]. In this case, the direct production of 3-phosphoglycerate is advantageous as it reduces significantly the amount of phosphoglyceromutase needed to be expressed.

The methodology we describe here, of using a metabolic assay with an easily readable growth output, is a useful tool to decipher the *in vivo* specificity of enzymes (*e*.*g*., see ref. [19]). The strategy has some drawbacks, *e*.*g*., enzyme promiscuous activity can results in false a positive result, especially at high expression levels of the enzyme in question. Yet, if one carefully controls for such effects, the metabolic selection strategy can clearly identify the substrates as well as products of various different enzymes. For example, to elucidate whether an enzyme is a decarboxylating or a non-decarboxylating malate dehydrogenase [20], one can apply a metabolic assay involving two strains. In the first strain all endogenous malate dehydrogenase and malic enzyme variants are deleted, together with the anaplerotic and cataplerotic enzymes. Upon overexpression of reversible PEP carboxykinase [21] and the enzyme in question, growth will be established on glutamine as a sole carbon source [22] if and only if the enzyme in question is indeed a malate dehydrogenase, whether decarboxylating or not. The second strain is identical to the first one with an additional deletion of PEP synthetase. This strain, also expressing PEP carboxykinase and the enzyme in question, would be able to grow on glutamine only if the malate dehydrogenase is non-decarboxylating, such that oxaloacetate can accumulate and be converted to PEP and other gluconeogenic intermediates. As demonstrated in this study, the main advantage of a metabolic assay strategy is that it does not rely on *in vitro* methods performed under changing artificial conditions that can lead to contradicting results. Rather, this approach tests the enzyme under physiological conditions and thus can undisputedly assign specificities in a simple and highly reproducible manner.

## Materials and Methods

### Cloning

*E*. *coli*’s *garK* and *glxK* genes were amplified directly from an *E*. *coli* K12 Mg1665 strain. The gene encoding for At glyK was amplified from *Arabidopsis thaliana*’s cDNA library. The primers used are given below. Cloning was done using the “no background” cloning method we previously developed [23]; the glycerate kinase genes were attached to RBS ‘E’ within the Pniv plasmid, and then inserted to a Ptac plasmid as described in details in ref. [23]. The EcoRI and SalI restriction sites that exist within the endogenous *glxK* and *Arabidopsis*‘s *glyK* genes were removed using a silent point mutation via the overhang extension procedure (primers used are given below) [24].

Gene deletions were performed using a standard P1 phage transduction [25]; all donor strains were taken from Keio collection [26]. We used PCP20 to mediate a flippase catalyzed excision of the antibiotic-resistance [27], thereby enabling further gene deletions using the same resistance marker.

Primers:

**Table:** 

Ec garK Forward	ATGCATCATCACCATCACCACGCGTATTGCAATCCGGGCCTGGAATC
Ec garK Reverse	CTCTTACGTGCCCGATCAACGCTAGCTTACCCCGCGTTGCGCATTCCAATCG
Ec glxK Forward	ATGCATCATCACCATCACCACAAGATTGTCATTGCGCCAGACTC
Ec glxK Reverse	CTCTTACGTGCCCGATCAACGCTAGCTTATTTTTAATTCCCTGACCTATTTTAATGGCG
Ec glxK C435G Forward	GTGCGACGGTTGACGGCGGTATGGGCATGG
Ec glxK C435G Reverse	CCATGCCCATACCGCCGTCAACCGTCGCAC
At glyK Forward	ATGCATCATCACCATCACCACTCTTCTTATTTATCCTCCAAGCTT
At glyK Reverse	CTCTTACGTGCCCGATCAACGCTAGCTTAGTTTGCGAGTATCGGGTTCCTTTC
At glyK C314T Forward	TTTTGAATTTATATGCTCGGGTCCTCTCGT
At glyK C314T Reverse	ACGAGAGGACCCGAGCATATAAATTCAAAA

### Growth assays

The different strains were grown overnight in 5 ml LB media containing Kanamycin (50mg/L) and chloramphenicol (30mg/L). Following OD measurements, we centrifuged ~10^9^ cells for 1 min at 9,500 g. The pellets were re-suspended in 1 ml of M9 medium, from which 10 μl were dispensed into a 96-multiwell plate. Each well also contained 200 μl of M9 medium supplemented with 0.2% appropriate carbon sources, as well as antibiotics (kanamycin 50mg/L and chloramphenicol 30mg/L). Notably, by taking ≈10^9^ cells for the initial centrifuge, we made sure that the initial OD in each well is ≈0.05.

The plates were cultivated within a LiCONiC incubator shaker at 37°c, 100% humidity and ambient gas composition. Every 90 minutes plates were automatically carried by a robotic arm (Evoware II, Tecan) to a plate reader (Infinite M200-pro, Tecan), in which OD (600nm) measurements were taken.

We picked several clones of each transformation for growth experiments. Different clones of the same strains were found to share the same qualitative growth phenotype, although with somewhat different growth yield and dynamics.
